# Practice patterns of hepatobiliary surgery within the military

**DOI:** 10.1007/s00464-023-10150-6

**Published:** 2023-07-06

**Authors:** Camille R. Suydam, Marcos C. Aranda, Thomas A. O’Hara, Fred C. Kobylarz, Joy N. Liang, Bradley Bandera

**Affiliations:** Department of Surgery, Eisenhower Army Medical Center, Fort Gordon, USA

**Keywords:** Centralization, Hepatobiliary, Military

## Abstract

**Background:**

The purpose of this study is to evaluate the trends of hepatobiliary surgeries performed at military hospitals and to discuss potential implications on resident training and military readiness. While there is data to suggest centralization of surgical specialty services leads to improved patient outcomes, the military does not currently have a specific centralization policy. Implementation of such a policy could potentially impact resident training and readiness of military surgeons. Even in the absence of such a policy, there may still be a trend toward centralization of more complex surgeries like hepatobiliary surgeries. The present study evaluates the numbers and types of hepatobiliary procedures performed at military hospitals.

**Methods:**

This study is a retrospective review of de-identified data from Military Health System Mart (M2) from 2014 to 2020. The M2 database contains patient data from all Defense Health Agency treatment facilities, encompassing all branches of the United States Military. Variables collected include number and types of hepatobiliary procedures performed and patient demographics. The primary endpoint was the number and type of surgery for each medical facility. Linear regression was used to evaluate significant trends in numbers of surgeries over time.

**Results:**

Fifty-five military hospitals performed hepatobiliary surgeries from 2014 to 2020. A total of 1,087 hepatobiliary surgeries were performed during this time; cholecystectomies, percutaneous procedures, and endoscopic procedures were excluded*.* There was no significant decrease in overall case volume. The most commonly performed hepatobiliary surgery was “unlisted laparoscopic liver procedure.” The military training facility with the most hepatobiliary cases was Brooke Army Medical Center.

**Conclusion:**

The number of hepatobiliary surgeries performed in military hospitals has not significantly decreased over the years 2014–2020, despite a national trend toward centralization. Centralization of hepatobiliary surgeries in the future may impact residency training as well as military medical readiness.

The concept of centralization is an important one in the discussion of delivering high quality health care. The rationale for centralization is that high-volume centers have better patient outcomes than do low-volume centers, and that this has been demonstrated in multiple studies [1–6]. This is likely due not only to surgeon case volume, but also experience levels of other services (i.e., anesthesiology team, critical care, nursing staff) as well as hospital resources [7–9]. While centralization is becoming increasingly more common, formal criteria for which surgeries warrant centralization as well as what qualifies a center to be considered “high-volume,” are yet to be established [7, 10–13].

Due to its complex nature and potential complications, hepatobiliary surgery is one of the surgical subspecialties in which patients may benefit from centralization. Multiple studies have found improved patient outcomes in regards to morbidity, mortality, and hospital stay after hepatobiliary surgery when performed at centers with high case volume [14–29], though the definition of “high volume” does vary across these studies. In one retrospective review of liver resections performed in the United States from 2002 to 2011, Idrees et al. found the overall in-hospital mortality was 2.2% and the incidence of major complication was 14.9%; the most common complications were surgical site infection (3.8%), hemorrhage (3.3%), renal failure (2.8%), and respiratory failure (2.7%). When comparing centers that performed fewer than 150 cases per year to centers that performed 150 cases or more per year, in-hospital mortality and major complication rates were significantly lower at the higher volume centers (1.3% vs 2.6% and 14.3% vs 15.4%, respectively) [30].

In addition to the technical expertise and hospital staff experience that contribute to decreased morbidity and mortality rates after hepatobiliary surgery, higher volume centers may better manage patients with major postoperative complications [9, 25, 31]. Furthermore, higher volume centers may also better delineate those patients that would benefit from early surgical intervention (i.e., in the case of metastatic disease to the liver or in the case of incidentally found gallbladder carcinoma) [32–34].

While centralization clearly does have its benefits, it is important to consider the negative effects centralization may have on patients, resident training, and military surgeon readiness. For example, centralization may make access to care difficult for certain patients based on socioeconomic status and/or geographic location. Regarding resident training, centralization has been cited as a possible reason for the decreasing number of hepatobiliary cases that general surgery residents are graduating with [35]. It may also be contributing to the decreased number of military-relevant cases that military general surgeons are performing, overall impacting their mission readiness [36, 37].

The purpose of this study is to evaluate and describe the patterns in hepatobiliary surgeries performed at military hospitals, and to discuss the implications these numbers may have on resident training and military surgeon readiness. We hypothesized that a small group of military treatment facilities perform the majority of procedures, and that there would be a downward trend in the overall number of hepatobiliary surgeries being performed at military facilities over the studied time period.

## Methods

We used Military Health System Mart (M2), a data collection system specific to the military medical record, to collect data from the outpatient setting from 2014 to 2020. Prior to accessing this anonymized data, the Dwight David Eisenhower Army Medical Center Institutional Review Board deemed this analysis exempt from review with written consent not necessary due to the nature of the study.

The outpatient electronic medical record for Military Treatment Facilities (MTFs), called the Armed Forces Health Longitudinal Technology Application (AHLTA), was used to analyze the number of hepatobiliary surgeries performed in MTFs from the years 2014 to 2020, as well as the demographics of the patients that underwent hepatobiliary surgery. Hepatobiliary surgeries analyzed were determined by Current Procedural Terminology (CPT) codes, and included hepatotomy (47010), liver wedge resection (47100), partial liver lobectomy (47120), trisegmentectomy/extensive removal of the liver (47122), total left lobe resection (47125), total right lobe resection (47130), allotransplant of liver (47135), surgery for a liver lesion (47300), repair liver wound (47350, 47360, 47361, 47362), laparoscopic ablation of tumor with radiofrequency ablation (47370), laparoscopic ablation of liver tumor with cryotherapy (47371), unlisted laparoscopic procedure of liver (47379), open ablation of liver tumor with radiofrequency ablation (47380), open ablation of liver tumor with cryotherapy (47381), liver surgery procedure (47399), incision of liver duct (47400), incision of bile duct (47420, 47425), incise bile duct sphincter (47460), bile duct revision (47701), excision of bile duct tumor (47711), excision of bile duct cyst (47715), fusion of bile ducts and bowel (47760, 47780, 47785), fuse liver ducts and bowel/intestine (47765, 47802), reconstruction of bile ducts (47800), suture bile duct injury (47900), and bile tract surgery procedure (47999). CPT codes that included cholecystectomies with or without cholangiography and with or without common bile duct exploration were excluded in the analysis (47562, 47563, 47564, 47600, 47605, 47610, 47612, 47620). These were excluded due to cholecystectomies being common procedures that would not typically be considered for referral to a specialty center. Endoscopic and percutaneous procedures were also excluded, as the purpose of this study was to evaluate trends in surgical procedures specifically.

The demographic information analyzed included gender, age, race/ethnicity, branch of military service, sponsor relationship, and facility. Because the analyzed data was obtained from the outpatient record, there was no documentation on inpatient complications. In 2017, Madigan army medical center (MAMC) began using an alternative outpatient medical record and thus some of their data from late 2017 through 2020 was unavailable.

Linear regression was used to compare the overall number of hepatobiliary surgeries performed over time. Further sub-analysis was performed using linear regression to compare the number of hepatobiliary surgeries performed by each branch of service (Army, Navy, and Air Force); the total number of surgeries performed at the four highest volume centers (Naval Medical Center Portsmouth, Madigan Army Medical Center, Brooke Army Medical Center, and Walter Reed National Military Medical Center); the total number of hepatobiliary surgeries excluding “unlisted laparoscopic liver procedure” (CPT code 47379); the total number of hepatic surgeries (CPT codes 47010–47400); and the total number biliary surgeries (47420–47999). Results were considered statistically significant if *p* < 0.05.

## Results

**Fig. 1:**
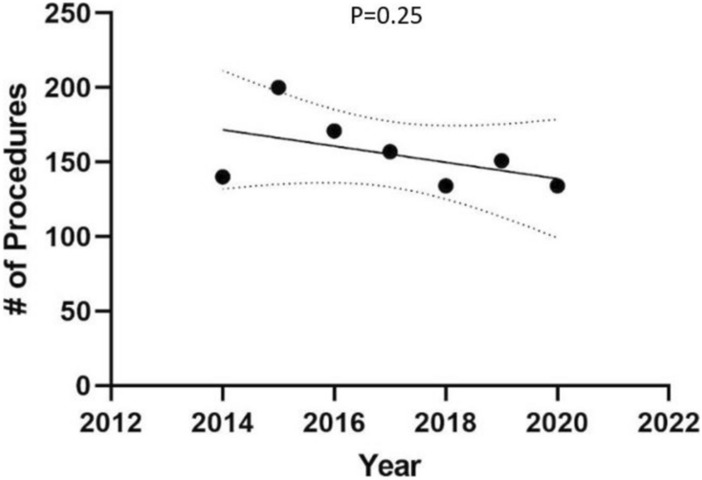
Total number of hepatobiliary surgeries from 2014 to 2020

The majority of patients were white, female, and between the ages of 60 and 69 (Table 1). Fifty-five military hospitals performed the hepatobiliary procedures analyzed from 2014 to 2020. There were a total of 1,087 hepatobiliary surgeries performed. The most surgeries were performed in the year 2015 (200), and the least were performed in the years 2018 and 2020 (134 each year). There was no significant decrease in the number of surgeries performed from 2014 to 2020 (*p* = 0.25) (Fig. 1).

**Table 1:** Patient demographics

Demographic	Category	Number (%)
Sex	Male	520 (47.8)
	Female	567 (52.2)
Age	< 20	27 (2.5)
	20–29	126 (11.6)
	30–39	146 (13.4)
	40–49	172 (15.8)
	50–59	224 (20.3)
	60–69	265 (24.3)
	70–79	85 (7.8)
	80–89	38 (3.5)
	> 89	4 (0.4)
Race	White	377 (34.7)
	Black	126 (11.6)
	Asian or Pacific Islander	69 (6.3)
	American Indian or Alaskan Native	2 (0.2)
	Other	287 (26.4)
	Unavailable	226 (20.8)
Sponsor Status	Active Duty	115 (10.6)
	Active Duty Family	155 (14.3)
	Dependent of Guard/Reserves on Active Duty	1 (0.1)
	Guard/Reserve on Active Duty	21 (1.9)
	Inactive Guard/Reserve	3 (0.3)
	Retiree Family	262 (24.1)
	Retirees	272 (24.1)
	Survivor	42 (3.9)
	Other	214 (19.7)
	Unlisted	2 (0.2)
Branch of Service	Army	404 (37.2)
	Air Force	220 (20.2)
	Navy	183 (16.8)
	Marines	40 (3.7)
	Coast Guard	13 (1.2)
	Unlisted	224 (20.6)
	PHS	3 (0.3)

**Fig. 2:**
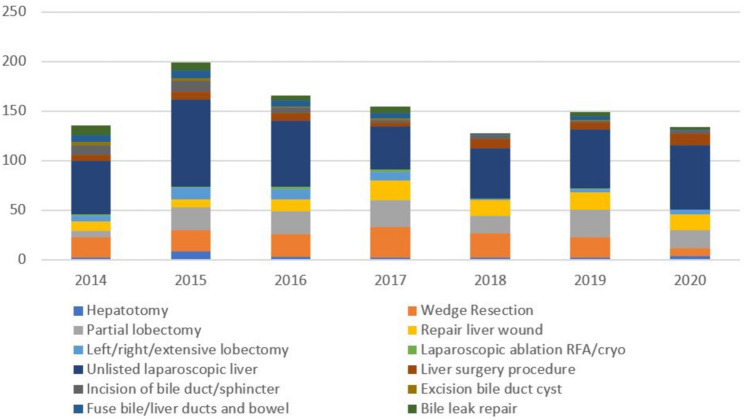
Hepatobiliary surgery by case. For simplicity, similar surgeries are grouped together and surgeries with < 10 cases are not included. See Table 2 for detailed list of surgeries

The most frequently performed hepatobiliary surgery was “unlisted laparoscopic liver procedure” (428), followed by “liver wedge resection” (150), “partial liver lobectomy” (142), and “repair liver wound” (99). There were no “allotransplantation of the liver” and “open ablation of tumor with cryotherapy” procedures performed. There was a single “excision of bile duct tumor” performed (Table 2; Fig. 2).

**Table 2:** Number of surgeries by year

Procedure	2014	2015	2016	2017	2018	2019	2020	Total (%)
Hepatotomy	2	9	3	2	2	2	4	24 (2.2)
Liver wedge resection	21	21	23	31	25	21	8	150 (13.8)
Partial liver lobectomy	6	23	23	27	17	28	18	142 (13.1)
Trisegmentectomy/extensive removal of liver	1	2	5	2	0	2	2	14 (1.3)
Total left lobe resection	3	6	1	3	1	0	1	15 (1.4)
Total right lobe resection	1	4	4	4	0	1	2	16 (1.5)
Allotransplant of liver	0	0	0	0	0	0	0	0 (0)
Surgery for liver lesion	2	0	2	2	2	0	0	8 (0.7)
Repair liver wound	10	8	12	20	16	17	16	99 (9.1)
Laparoscopic ablation of liver tumor with RFA	2	0	3	2	1	0	0	8 (0.7)
Laparoscopic ablation of liver tumor with cryotherapy	0	1	0	0	0	1	0	2 (0.2)
Unlisted laparoscopic procedure, liver	54	88	66	44	51	60	65	428 (39.4)
Open ablation of liver tumor with RFA	1	0	1	0	1	0	0	3 (0.3)
Open ablation of liver with cryotherapy	0	0	0	0	0	0	0	0 (0)
Liver surgery procedure	6	8	8	4	9	7	12	54 (5.0)
Incision of bile duct	6	10	6	2	4	1	3	32 (2.9)
Incise bile duct sphincter	4	1	0	0	0	0	0	5 (0.5)
Incision of liver duct	0	0	2	0	2	1	0	5 (0.5)
Bile duct revision	0	1	0	0	0	0	0	1 (0.1)
Excision of bile duct tumor	0	0	0	0	0	1	0	1 (0.1)
Excision of bile duct cyst	3	2	1	2	0	1	0	9 (0.8)
Fuse bile ducts and bowel	6	8	6	4	1	4	1	30 (2.8)
Hepaticojejunostomy	1	0	0	1	0	0	0	2 (0.2)
Reconstruction of bile ducts	1	0	0	0	0	0	0	1 (0.1)
Suture bile duct injury	0	0	0	0	1	0	0	1 (0.1)
Bile leak repair	10	8	5	7	1	4	2	37 (3.4)
Totals (%)	140	200	171	157	134	151	134	1087

Sixty-six percent of procedures were performed at Army facilities, 17% at Navy facilities, 11% at Air Force facilities, and 6% at joint facilities. The military training facility with the most hepatobiliary cases was Brooke Army Medical Center (287 cases), followed by Madigan Army Medical Center (108 cases), Naval Medical Center Portsmouth (79 cases), and Walter Reed National Military Medical Center (55 cases). Combined, these four MTFs performed 49% of all hepatobiliary surgeries. Twenty-five of the 55 military facilities performed fewer than five procedures over the seven-year course, and 32 preformed fewer than ten (Table 3).

**Table 3:** Number of surgeries by facility and number of surgeries by military service (bold)

Facility	2014	2015	2016	2017	2018	2019	2020	Total (%)
ARMY	**87**	**120**	**115**	**100**	**86**	**118**	**92**	**720 (66.2)**
Brooke Army Military Medical Center	40	48	36	40	32	58	33	287 (26.4)
Bassett Army Community Hospital	2	5	3	3	2	3	4	22 (2.0)
Madigan Army Medical Center	14	25	26	17	8	9	9	108 (9.9)
Puyallup Community Medical Home—Madigan Army Medical Center	0	0	0	0	0	0	1	1 (0.1)
William Beaumont Army Medical Center	8	6	4	12	2	14	4	50 (4.6)
Tripler Army Medical Center	4	11	8	6	5	9	6	49 (4.5)
Dwight D Eisenhower Army Medical Center	1	4	2	1	2	4	14	28 (2.6)
Womack Army Medical Center	3	1	6	3	3	5	1	22 (2.0)
Ft Belvoir Community Hospital	1	4	4	5	0	1	4	19 (1.7)
Brian D Allgood Army Community Hospital	1	0	1	0	0	1	4	7 (0.6)
Weed Army Community Hospital	0	0	0	0	2	2	0	4 (0.4)
Evans Army Community Hospital	2	5	4	0	2	2	2	17 (1.6)
Bayne-Jones Army Community Hospital	1	0	0	0	0	0	0	1 (0.1)
Martin Army Community Hospital	0	0	4	0	0	0	0	4 (0.4)
Leonard Wood Army Community Hospital	0	3	4	5	9	0	4	25 (2.3)
McDonald Army Health Center	0	0	0	0	2	0	0	2 (0.2)
Blanchfield Army Community Hospital	2	2	1	0	7	1	3	16 (1.5)
Landstuhl Regional Medical Center	2	2	5	1	2	2	3	17 (1.6)
Keller Army Community hospital	0	0	1	1	3	2	0	7 (0.6)
Irwin Army Community Hospital	0	1	0	0	3	0	0	4 (0.4)
Winn Army Community Hospital	3	0	2	0	0	1	0	6 (0.6)
Ireland Army Health Clinic	0	1	0	0	0	0	0	1 (0.1)
Reynolds Army Health Clinic	2	0	0	0	0	0	0	2 (0.2)
Darnall Army Medical Center	1	4	4	6	2	4	0	21 (1.9)
NAVY	**34**	**47**	**23**	**22**	**18**	**18**	**22**	**186 (17.1)**
Naval Medical Center Portsmouth	19	22	7	9	7	7	8	79 (7.2)
Ascension St. Vincent’s	0	0	0	0	0	0	1	1 (0.1)
Naval Health Clinic New England	0	0	0	1	0	0	0	1 (0.1)
U.S. Naval Hospital Yokosuka	0	0	0	0	0	0	2	2 (0.2)
Naval Hospital Bremerton	2	0	0	0	0	0	0	2 (0.2)
Naval Hospital Jacksonville	1	2	2	2	1	2	1	11 (1.0)
Naval Medical Center San Diego	5	12	9	4	3	3	6	42 (3.9)
Naval Hospital Camp Pendleton	0	2	0	2	1	0	0	5 (0.5)
Naval Medical Center Camp Lejeune	0	0	1	2	0	3	4	10 (0.9)
U.S. Naval Hospital Guam	4	0	0	0	0	0	0	4 (0.4)
Naval Hospital Pensacola	2	9	2	2	4	2	0	21 (1.9)
U.S. Naval Hospital Naples	0	0	2	0	0	1	0	3 (0.3)
U.S. Naval Hospital Okinawa	1	1	0	0	2	0	0	4 (0.4)
Las Palmas?	0	1	0	0	0	0	0	1 (0.1)
AIR FORCE	**16**	**23**	**26**	**22**	**13**	**12**	**5**	**118 (10.9)**
Malcom Grow Medical Clinics and Surgery Center	0	0	0	0	2	0	1	3 (0.3)
Misawa AB 35^th^ medical Group	0	0	0	0	1	0	0	1 (0.1)
USAF 366^th^ Medical Group	0	1	0	0	0	0	0	1 (0.1)
Aviano AB 31^st^ Medical Group	0	0	0	2	0	0	0	2 (0.2)
Yokota AFB 374^th^ Medical Group	0	0	1	0	0	0	0	1 (0.1)
RAF Lakenheath Medical Hospital	0	2	0	0	0	0	0	2 (0.2)
10^th^ Medical Group Air Force Academy	1	0	0	0	0	0	0	1 (0.1)
Willford Hall Ambulatory Surgical Center	0	0	0	0	0	1	0	1 (0.1)
Langley AFB Hospital	0	1	0	0	0	2	0	3 (0.3)
Eglin Air Force Base Hospital	4	8	6	4	4	2	0	28 (2.6)
Mike O’Callaghan Military Medical Center	2	1	1	1	0	1	0	6 (0.6)
Keesler Medical Center	2	0	0	2	1	1	3	9 (0.8)
David Grant Air Force Medical Center	3	5	10	3	2	3	0	26 (2.4)
Wright-Patterson Medical Center	4	6	6	10	3	2	1	32 (2.9)
Banner Estrella Medical Center	0	0	2	0	0	0	0	2 (0.2)
JOINT	**3**	**5**	**7**	**13**	**17**	**3**	**15**	**63 (5.8)**
Walter Reed National Military Medical Center	0	5	7	12	13	3	15	55 (5.1)
Joint Base Elmendorf-Richardson	3	0	0	1	4	0	0	8 (0.7)
Totals	**140**	**200**	**171**	**157**	**134**	**151**	**134**	**1087**

**Fig. 3:**
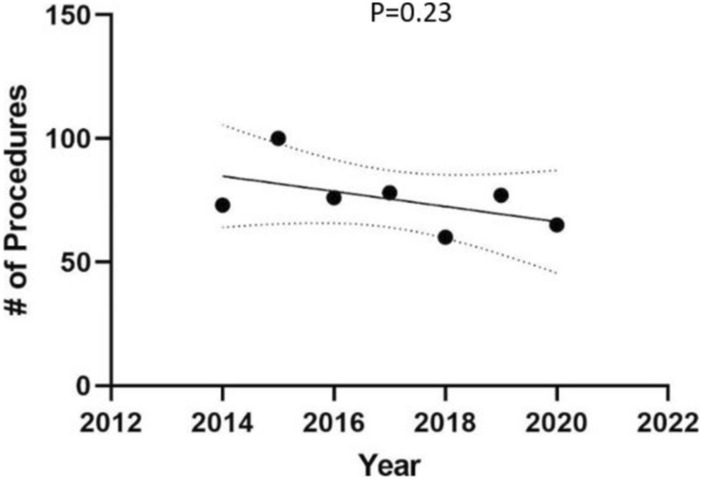
Number of hepatobiliary surgeries performed at four highest volume military centers

**Fig. 4:**
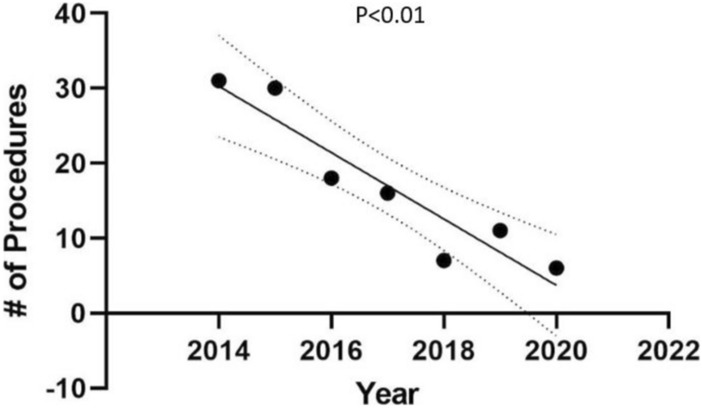
Total number of biliary surgeries performed from 2014 to 2020

On sub-analysis by branch of service, there was no significant decrease in the number of surgeries performed from the year 2014 to 2020 in the Army (*p* = 0.84), Navy (*p* = 0.07), or Air Force (*p* = 0.07). There was also no significant change in the number of surgeries performed over these years at the four highest volume centers (*p* = 0.23) (Fig. 3). There was no significant difference in the number of hepatobiliary surgeries performed after exclusion of “unlisted laparoscopic liver procedure” (*p* = 0.21). There was no significant decrease over these years in the number of total hepatic surgeries performed (0.81). There was a significant decrease in the number of biliary surgeries performed over these years (*p* < 0.01) (Fig. 4).

## Discussion

Our results showed that the number of hepatobiliary surgeries being performed across all military hospitals did not significantly decrease from the year 2014 to the year 2020. While this was unexpected, there are multiple factors that may have contributed to this finding. One reason that we may not have seen the centralization trend that we were expecting is that our data did not capture the numbers of surgeries performed prior to 2014. While certainly more prominent in recent years, centralization is not a new concept and may have occurred prior to 2014, especially among those military hospitals that performed fewer than ten hepatobiliary surgeries over the seven-year period. In addition, the year 2014 did appear to be an outlier; interestingly, Walter Reed had zero hepatobiliary cases during this year. The year 2014 was also the only year that used the ICD-9 codes only, since the newer ICD-10 system was implemented in 2015 (Fig. 4).

Since “unlisted laparoscopic liver procedure” was the most frequently performed surgery and may comprised of less technically complex cases (such as liver biopsies), we evaluated the trend in the number of surgeries after excluding it; however, there was still no significant decrease over the time period. This may be due to the fact that the other most frequently performed hepatobiliary surgeries were liver wedge resections, partial lobectomies, and repair of liver wounds which are also some of the less technically complex surgeries included in the analysis. The relative abundance of these less complex cases may help explain why there has not been a significant decrease in the number of hepatobiliary cases overall from 2014 to 2020. This is supported by our finding that there was a significant decrease in the number of biliary surgeries alone on sub-analysis, of which many would be considered more technically complex cases as less complex biliary surgeries (i.e., cholecystectomies) were intentionally excluded in the analysis. Of note, there was only one bile duct tumor excision performed over the seven-year period. Whether this is a reflection of the rarity of these procedures in the study population or that these highly complex biliary cases are being referred to a high-volume center is unclear, although the latter is likely at least in part true. Idrees et al. found that while not formally centralized in the civilian sector either, the proportion of cholangiocarcinoma resections done at high-volume centers increased from 25% in 2004 to 44% in 2015 [16]. Additionally, there were no liver transplants performed at the selected MTFs during the study period. This is not surprising, as the military no longer offers a transplant fellowship and instead relies on civilian facilities to provide these services [38].

Our results showed that the majority of hepatobiliary surgeries were performed at a select number of military hospitals. Nearly half (49%) of all queried cases were performed at only four of the 55 centers: Brooke Army Medical Center, Madigan Army Medical Center, Naval Medical Center Portsmouth, and Walter Reed National Military Medical Center. The majority of military facilities (32 of 55) performed fewer than ten hepatobiliary surgeries over the seven-year period. These findings suggest that centralization may be occurring within the military itself despite there being no formal decree. Upon sub-analysis, there was no significant change in the number of surgeries performed at these four centers from the year 2014–2020. While an increase in the number of surgeries would be expected if centralization was indeed occurring in the military, as discussed previously, centralization may have been occurring prior to 2014 and thus not captured by the present dataset. While not captured by our demographic data, it would be interesting to know if the patient population that underwent surgery at these higher volume centers was comprised mostly of patients local to the area or if there was a significant portion that traveled from other geographic locations to undergo surgery.

If our data is an accurate reflection of centralization trends, it is important to consider if a formal centralization policy should be adopted given the potential benefits on patient outcomes. There are many factors and challenges to consider in this discussion. One issue to consider is cost. There is mixed data regarding the financial implications of centralization, with some studies arguing it leads to overall decreased costs while others argue the opposite [4, 10, 18, 26, 30]. Other potential challenges include lack of hepatobiliary specialists; long wait times for patients; and access barriers for patients due to geographic, socioeconomic, cultural, and other patient-specific factors [10]. These barriers are especially important to consider in situations where expedited care is needed, such as in patients with a diagnosis of cancer.

While there is robust evidence that patient outcomes improve after hepatobiliary surgery at a high-volume center, it has been argued these improved outcomes may in part be due to socioeconomic factors. Eppsteiner et al. found that patients receiving care at high-volume centers were more likely to have private insurance and be admitted electively. However, they still found that liver resection performed by both high volume surgeons at high-volume centers was independently associated with lower in-hospital mortality [39]. It has also been argued that while patients requiring more complex hepatobiliary surgeries may be better served at a high-volume center, small accessible hepatic lesions may be just as well managed by their low-volume counterparts [40].

Another aspect that should be considered when discussing a formal centralization policy is the implications on surgical resident training. Diaz et al. found that there has been a decrease in the mean number of complex hepato-pancreato-biliary general cases that general surgery residents graduate with, as well as an increase in the variability of case numbers among residents; the authors speculate that recent trends toward centralization are in part contributing to this change [35].

Finally, there are military-specific considerations when it comes to centralization. One way to measure military surgeon readiness is in Knowledge, Skills, and Ability (KSA) points; this metric is designed to quantify readiness based on the number of KSA-generating procedures a military surgeon has completed. Dalton et al. found the number of KSA-generating procedures performed at military hospitals dropped by 25% from the year 2015 to 2019, with a resultant decrease in the number of military general surgeons meeting the KSA metric readiness threshold [36]. Similarly, another study found an increasingly higher number of surgeries deemed to be relevant for deployed surgeons (to include hepatectomy) were being increasingly referred to non-military specialty centers from 2005 to 2019 [37].

## Limitations

One of our limitations is that we may have incomplete data for Madigan Army Medical Center, as from 2017 to 2020 they transitioned to a new electronic medical record. We also did not capture the trends in hepatobiliary surgery numbers prior to 2014, which if included may make a stronger or weaker case for the occurrence of centralization of hepatobiliary surgery in the military. Additionally, the data we collected was dependent on accurate coding of procedures; of note, the most common surgery performed was “unspecified laparoscopic liver procedure” which may have captured procedures we would have otherwise excluded. Our demographic data was limited in that we did not have geographic location of patients, which would have been helpful in determining if patients are traveling or being transferred from lower volume military facilities to higher volume military facilities.

## Conclusion

The number of hepatobiliary surgeries performed in military hospitals has not significantly decreased over the years 2014–2020, however, there may be a trend of centralization within the military despite no formal protocol. While centralization has been found to lead to improved patient outcomes, centralization to high-volume centers outside the military would have a significant impact on military residency training and readiness without developing formal training agreements with civilian centers.
